# Investigation of a Scabies Outbreak in Drought-Affected Areas in Ethiopia

**DOI:** 10.3390/tropicalmed3040114

**Published:** 2018-10-29

**Authors:** Wendemagegn Enbiale, Ashenafi Ayalew

**Affiliations:** 1 Dermatology and Venerology, Bahir Dar University, P.O. Box 1996, Bahir Dar, Ethiopia; 2 Amhara Regional Health Bureau, P.O. Box 744, Amhara, Ethiopia; ashunets@gmail.com

**Keywords:** scabies, outbreak, drought, emergency state

## Abstract

The impact of the severe drought in Ethiopia, attributed to El Niño weather conditions, has led to high levels of malnutrition that have, in turn, increased the potential for disease outbreaks. In 2015, Ethiopia faced a scabies outbreak in drought-affected areas where there was a shortage of safe water for drinking and personal hygiene. Following a house-to-house census to assess the prevalence of scabies, a detailed study was conducted looking at the disease burden. Following the outbreak report, training was provided on scabies identification and management for zonal and district health officials from administrative districts affected by the drought (nutritional hot-spot *woredas*). The training was cascaded down to the health extension workers in the affected areas. Screening and management guidelines and protocols were also distributed. House-to-house data collection was undertaken by 450 health extension workers (HEWs) to assess the prevalence of scabies. The HEWs used a simplified reporting tool. Subsequently, data were collected and validated in two zones and six *woredas* from 474 participants who had been diagnosed with scabies using a standardized questionnaire. This was designed to look at the specificity of the diagnosis of scabies, age distribution, severity, duration of illness, secondary infection and other sociodemographic variables as preparation for mass drug administration (MDA). The HEWs screened 1,125,770 people in the 68 districts in Amhara Region and a total of 379,000 confirmed cases of scabies was identified. The prevalence in the different districts ranged from 2% to 67% and the median was 33.5% [interquartile range (IQR) 19–48%]. 49% of cases were school-aged children. The detailed study of 474 individuals who were recorded as scabies cases revealed that the specificity of the diagnosis of scabies by the HEWs was 98.3%. The mean duration of illness was 5 months (SD of ± 2.8). One third of patients were recorded as having severe illness, 75.1% of cases had affected family members, and 30% of affected children were noted to have secondary bacterial infection. Eleven percent of the students had discontinued school due to scabies or/and drought and 85% of these had secondary bacterial infection. These community-based data serve as reliable proxy indicators for community-based burden assessment of the scabies epidemic. This study will also provide a good basis for advocating the use of a community-level clinical diagnostic scheme for scabies using an algorithm with a simple combination of signs and symptoms in resource-poor settings.

## 1. Background

Scabies is a common public health problem, globally affecting about 200 million people. It is a particular problem where there is social disruption, overcrowding, and where personal hygiene is poor. Immunosuppression, poor nutritional status, and dementia are also risk factors for scabies [1,2,3]. Natural disasters, war, and poverty lead to overcrowding and have been associated with increased rates of transmission [4].

Scabies is caused by an ecto-parasitic infestation of the skin by the human itch mite, *Sarcoptes scabiei* var. *hominis* [1]. It usually spreads by direct, prolonged, skin-to-skin contact with an infested individual. The main effect of scabies is debilitating itching, leading to scratching, which in turn is followed by breakdown of the barrier function of the skin and complications due to bacterial infection, ranging from impetigo, abscesses, and cellulitis, to more serious conditions such as septicemia and glomerulonephritis, leading to renal failure and rheumatic heart disease [1,4].

### 1.1. Drought

The data from The International Disaster Database indicate that in Africa more than 40 million people were affected by drought in 2015–2016. Following the 2015–2016 El-Niño event, which affected many countries globally, Ethiopia experienced drought and extreme water shortage across large parts of the country. This has further limited access to water for personal hygiene and basic sanitation for many individuals, especially those in rural communities, leading to a great increase in the risk of communicable diseases like scabies and diarrheal diseases [5].

Most definitions of drought describe it as a prolonged period of abnormally low rainfall, leading to a shortage of water. The effects of drought are critically dependent on both context and vulnerability of the underlying population. The development and severity of the drought depends on the background level of water use, which may also influence the timing of the onset, duration, and end of the drought, as well as the social, economic, and administrative infrastructures which address the consequences of water deficit. The impact on health is particularly dependent on the socio-economic environment that, in turn, has a direct impact on the resilience of the population. Poor health, poverty, and conflict are additional contributing factors that exacerbate the impact of drought [6,7].

### 1.2. Government Priorities/Political Setting

Following official acknowledgment by the Ethiopian government of a food crisis in July 2015, the Federal Ministry of Health established a command post spearheaded by the Public Health Emergency Management (PHEM) directorate. In September 2015, the Amhara Regional State PHEM announced the first report of a scabies epidemic [8]. To validate the diagnosis, a team of field epidemiologists and a dermatologist visited three of the areas where the claim had emerged. The experts collected data from three health centers and six health posts in addition to visiting households that had been previously diagnosed with scabies at local health facilities. The experts concluded that scabies was the major active public health problem affecting the community, with the status of an outbreak [9].

Prior to September 2015, the regular review of drought-related public health emergencies, in both federal and regional PHEM disease surveillance systems, focused solely on malnutrition, diarrheal diseases, measles, malaria, and meningitis; however, there was no consideration of skin-related conditions. A recent literature review by Anderson and Davies has shown that El Niño has been associated with increases in the occurrence of sun-related skin diseases and certain vector-borne and waterborne diseases [10]. Despite outbreak reports in some parts of the country [8], scabies was not included in either the list of reportable diseases or the weekly reports.

After the first visit to some of the affected zones and a review of information regarding the burden of the problem, the inspection team advocated the inclusion of scabies as a separate item on the weekly surveillance list for the Federal Ministry of Health and Regional Health Bureau. Since October 2015, scabies has been included as one of the reportable diseases in the drought affected areas. The weekly PHEM surveillance reports revealed that scabies was becoming significantly more extensive than its more usual occurrence as sporadic clinical cases, and was now a public health concern affecting wider geographic areas and population groups, especially in the *woredas* most severely affected by drought and malnutrition. Hence, it required public mobilization and public health emergency interventions. The report revealed that the Amhara, Tigray, and Oromia regions had the highest burdens of scabies in Ethiopia.

The extent of the drought and the increased number of water-scarce *woredas* with limited access to water sanitation and hygiene (WASH) interventions, further worsened the spread of the disease and its severity among the vulnerable. From the November 2015 harvest season assessment, the number of nutritional hot-spot *woredas* increased to 429, involving a total population of nearly 49 million. That same month, the public health emergency management task force received a report that the number of severely affected *woredas* in Amhara, Tigray, and Oromia had reached 32, and despite the limited surveillance program for scabies, the estimated total number of cases reached more than 250,000 with prevalence of at least 15% in some districts [6].

In October 2015, following the national government elections, prominent officials at all levels of the administration attended an official meeting. The researchers used this as an opportunity to present the preliminary data of the burden of scabies and the recommended action to be taken by the relevant officials (zonal and *woreda* administrators, zonal and *woreda* health office officers, heads of the Regional Health Bureau, and other officials) and to advocate for region wide evidence generation in preparation for the intervention.

A systematic review of scabies prevalence studies published between 1985 and 2015, included only five African countries, highlighting the paucity of prevalence data in Africa [11]. This house-to-house census and validation study of scabies provides a unique opportunity to ascertain community prevalence. Such data is more reliable than health facility-based data which generally underestimates prevalence, as individuals within communities may not present with scabies (perhaps because it is ‘normalized’) or because there is under-diagnosis or lack of effective treatments at clinics [12].

## 2. Methods

The outbreak assessment developed in two stages.

### 2.1. Phase 1

**Figure 1:**
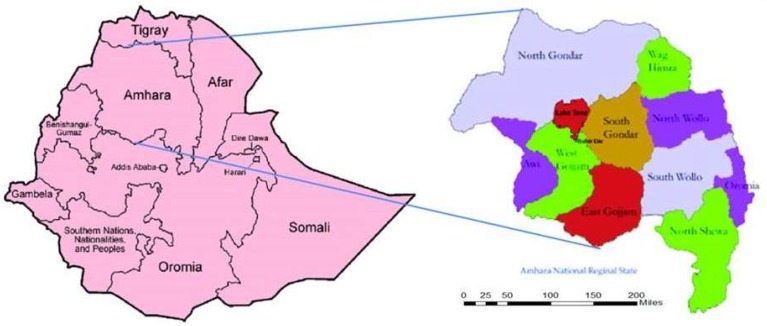
Amhara regional State, Ethiopia (2015/2016).

Following one-day face-to-face training on data recording, diagnosis, and management of scabies along with other relevant conditions for district health officials and the subsequent cascading of training to the front line care workers or health extension workers, a house-to-house census was undertaken in all the 68 hotspot *woredas* (districts) in the Amhara region (Figure 1).

A cross-sectional house-to-house census was performed, identifying scabies cases and contacts based on regional scabies identification and management guidelines in the Amhara region as preparation for the developing a scabies outbreak control program [7]. The census was performed in all households of the previously identified 68 hot-spot districts. The sites were selected by the Regional Health Bureau and PHEM team from the routine weekly report (districts in the region that have reported scabies). In each household, all family members were screened. The study period was from October to November 2015.

The census was conducted as part of the Regional Health Bureau screening in preparation for a community-based scabies control program. The local HEW, supervised by health officers/BSc nurses from nearby health centers, registered each resident by name, age, and sex and each resident was screened for scabies. Age groups were defined as follows: <2 years, 2 to 18 years, >18 years.

Each participant was examined by a HEW trained in scabies diagnosis and treatment. The diagnosis of scabies was based on the clinical case definition (Table 1). In addition to scabies screening, the census was used for the integrated assessment of pre-determined drought-related health problems (malnutrition, measles, diarrheal diseases, meningitis, malaria, and scabies). The screening of drought related pre-determined diseases by the health extension workers in relation to scabies had, up to this point, used only a limited set of information variables to identify cases of scabies and their contacts in each district.

The data were analyzed and results are presented using descriptive statistics. Scabies prevalence in the different age group and districts was calculated. In addition, statistical analysis was performed with SPSS 10.0 for Windows (SPSS Inc., Chicago, IL, USA). The median, interquartile range (IQR) and odds ratio (OR) were calculated and the *p*-value derived.

### 2.2. Phase 2

This study, performed between 16 October and 2 December 2015, was undertaken primarily to validate the data collected by the HEW and secondly to obtain an understanding of associated factors of the scabies epidemic. The study population was residents identified as cases in the house-to-house census. From the two zones, six districts with an estimated population of 600,000 were selected using clustered sampling techniques. The sample size was determined using a single proportion formula with consideration of the scabies median prevalence from the house-to-house census (33.5%) with a confidence level of 97% in the two zones. The reporting forms of the HEWs were used to select 474 cases. The inclusion criteria included those patients who have been identified as a case of scabies by the HEWs and the selection was performed with simple random sampling using the registration form. The team invited each patient to the village health post for a dermatology review including history, full body physical examination, and a standardized questionnaire which was completed by the dermatologist. The questionnaire included socio-demographic details of cases and additional variables such as presence of itching, severity of infestation, duration of illness and presence of secondary bacterial infection. The diagnosis of scabies was made clinically using the standardized case definition for the study (Table 1).

For clinical purposes, the severity of scabies was defined by the number of skin lesions and the presence or absence of crust. Mild: when the number of scabies associated skin lesions (papules, excoriation, and pustule) was less than or equal to ten. Moderate: The number of scabies associated skin lesions were between 10 and 50 lesions. Severe: when the number of scabies associated skin lesions were equal to or greater than 50 or the presence of scabies-associated crust. Secondary infection was defined by the presence of pustules and/or yellowish crusts.

The study received ethical approval on 14 September 2015 from the Regional Health Bureau as part of its operational research (reference number R.T.T/1/63107). A written support letter was received from the Regional Health Bureau to enlist the cooperation of the Zonal Health offices. Permission was subsequently also secured from the zonal and district health offices. The data collectors obtained verbal consent from each participant or his/her guardians. Written consent was obtained from patients who had images recorded.

The data was analyzed and results presented using descriptive statistics.

## 3. Results

### 3.1. Phase 1

**Figure 2:**
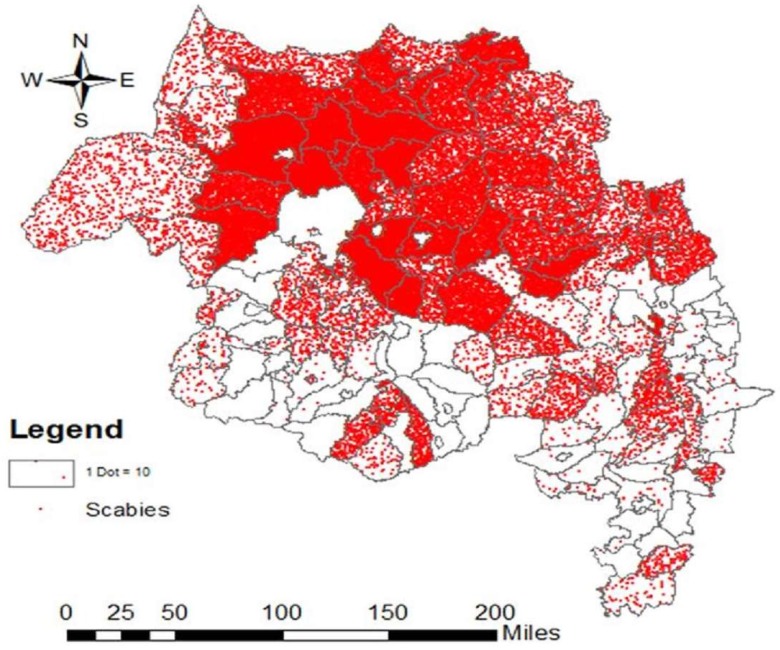
Scabies distribution in in Amhara Region, Ethiopia (2015–2016).

The field based house-to-house survey using HEWs in the Amhara Region screened 1,125,770 individuals from October to November 2015 in 68 districts (Figure 2). A total of 379,000 confirmed cases were found. Overall, 195,665 (51.6%) of the patients were female and (60%) of confirmed cases were below 18 years of age. Children and young adults under 18 years old were 2.5 times more at risk in developing scabies (Table 2).

The scabies prevalence in the 68 districts ranged from 2% to 67% with a median prevalence of 33.5 (IQR 19–48%).

### 3.2. Phase 2

**Figure 3:**
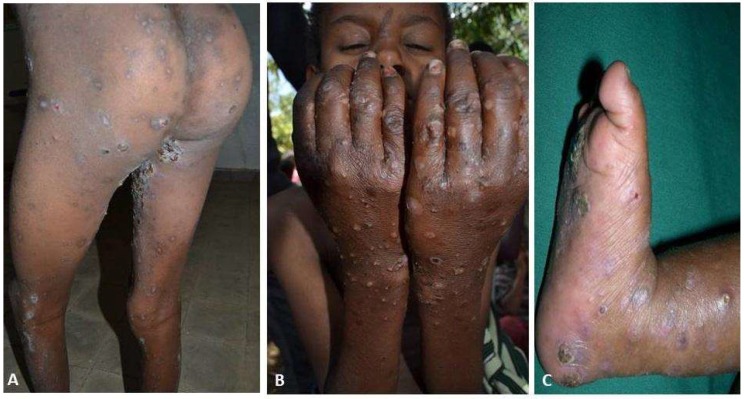
(**A**) Extensive crusting papules, excoriation with post-inflammatory hyperpigmentation in a 34-year-old female. (**B**) Pustules, crusts, and erosion in 12-year-old boy. (**C**) Pustules, crusts, and erosions in a 2-year-old female.

Of the 474 subjects enrolled in the ‘detailed’ study, 466 (98.3%) were confirmed to have scabies by a dermatologist (expert control group), which meant that the clinical diagnosis of the HEWs achieved a level of agreement with this control group of 98.3% (Figure 3). The index cases of the epidemic have been traced in both areas to the students in traditional church schools suggesting that these groups are important as sources of the infestation and in continued community transmission.

The majority (66.9%) of the scabies cases in this part of the study were in males, although the gender ratio varied with age. The proportion of males was by far the highest (113 or 87%) in those aged 18 years and above.

The median age of those affected was 10 years. The highest proportion of cases was found in those between 2 and 18 years old. 182 (39%) of the total number of cases were of school age and, of these, 63% were currently attending school, 15% never attended school, 11% had discontinued schooling prior to the drought and scabies outbreak and another 11% had discontinued their education as a result of scabies and the drought.

In the same validation study, all cases (466) had scabies skin lesions and all cases reported itching (itching and classic skin lesion are two case definitions used for scabies). The median number of lesions was found to be 10 to 49 (moderate). Approximately a quarter (116) of the cases had a history of a skin sore, of whom 94 (81%) had encrusted sores (Figure 3A). There were 14 (3%) cases with crusted scabies (Table 3).

The median duration of illness was 5 months, ranging from 0.5 to 12 months. Approximately one quarter (116) developed a secondary bacterial infection; the secondary bacterial infection rate was highest among those aged between 2 and 18 years (Table 4). On the other hand there was no significant difference in gender-specific bacterial infection rates (25% and 26% for females and males respectively). The bacterial infection rate among the school age group was 40% (Figure 3B). The rate was significantly higher 85% (95% CI of 79–91 and *p* < 0.001) among those who had dropped-out of school.

## 4. Discussion

This regional census documenting cases of scabies and the cross-sectional validation study for the intervention campaign, documents the burden of community-based scabies in Ethiopia. This survey was noteworthy in screening the highest number of cases ever reported in the literature and, at the same time, in documenting the largest number of scabies cases. Other cross-sectional community-based studies in relatively large populations have been reported: (1) in Malawi in a village with a population of 61,735 where the prevalence was 0.7%; and (2) in Cambodia in 13 villages with a population of 14,843 where the prevalence was 4.3% [13,14,15,16,17]. As a community-based study the median prevalence in our survey was much larger and the range much wider (2% to 67%). This is supported by previous reports that the prevalence of scabies in community-based surveys is frequently higher than the burden reported in the Global Burden of Disease study (GBD) or reports from routine clinic data [12,18].

Even though there is no evidence on a cause and effect association, the scabies outbreak occurred following the 2015–2016 El Nino drought. In contrast, the study by Andersen et al. reported scabies to be one of the dermatological problems with a significant decrease in incidence related to El Nino [10].

Hay et al., in their review of scabies in the developing world, noted that ‘the attack rate is probably equal between the sexes, and the differences in prevalence reported in some studies are probably attributable to confounding factors’, which was also confirmed in this large population census [19]. Seventy-three percent of infestations were registered in those under the age 18, which correlates with the impression that in developing countries, scabies is much more common among preschool children than adolescents and rates significantly decreased in mid-adulthood [20,21].

The burden of this scabies epidemic was compounded by the high rate of secondary bacterial infection, with 30% of school-age children presenting with mild to severe secondary bacterial infection, in agreement with another population based study in the Solomon Islands [22]. This survey will strengthen the data that show that scabies infestations are an important risk factor for bacterial infection of the skin [23,24].

The key conclusions are, firstly that this community based data will serve reliable proxy indicators for the burden of the scabies epidemic in communities. Secondly this study also reaffirmed the idea that community level clinical diagnosis of scabies by community health workers using a simplified diagnostic algorithm has the potential to address the scabies burden in resource poor settings [25,26]. Finally this study will also serve as a sizeable input for the WHO’s ‘global strategy for scabies control’ [27].

There are potential limitations in this study which are listed as follows:

1. The study used an aggregated report of the census with a limited data set (scabies case, contact, age, and sex) which could be a limitation of the study, especially for risk factor analysis.
2. In the house-to-house census of scabies cases, the examining HEWs had no other relevant experience apart from the one-day training described, which may have led to missed cases. The validation study focused only on specificity of the diagnosis.
3. Both in the census and in the validation study, the diagnosis of scabies and impetigo was made on the basis of clinical history and skin examination alone. Skin scrapings for direct microscopy or other confirmatory tests were not used.

Despite these limitations, the population survey and validation study have provided important new data about the extent of scabies in communities in northern Ethiopia and has registered the highest number of scabies cases ever reported in a single outbreak.

## Figures and Tables

**Table 1:** Clinical case definition of scabies and contact [7].

**Scabies**	Presence of itching with typical lesions on hands, inter-digital, and/or genitalia and/or itching and close contact with an individual who has itching or typical lesions in a typical distribution.
**Contact**	A contact is a person who does not fulfill the clinical criteria for infestation with scabies (above) or a person without signs and symptoms consistent with scabies, who has had direct contact (particularly prolonged, direct, skin-to-skin contact) with a suspected or confirmed case in the two months preceding the onset of scabies signs and symptoms in the index case

**Table 2:** Prevalence of scabies and bivariate analysis of demographic factors based on a house-to-house census in Amhara Region, Ethiopia (October 2015).

Variable	Total (*N*)	Affected (*n*)	Prevalence	OR *	*p* Value
Sex					
Female	585,400	195,665	33.4	Ref	Ref
Male	540,370	183,335	33.9	1.05	0.8
Total	1,125,770	379,000	33.7		
Age group (years) **					
<2	60,792	27,909	45.9	2.5	0.01
2 to 18	518,980	249,535	48.1	2.4	0.01
>18	545,998	101,556	18.6		

* Odds Ratio. ** Age groups were defined as follows: <2 years, 2 to 18 years, >18 years.

**Table 3:** Clinical signs and symptoms of scabies cases in the Amhara region, Ethiopia (2015).

Variables	*N* (%)
**Sign and symptom**	
Itching	466 (100%)
Classic scabies lesion	466 (100%)
Bacterial infection	116 (25%)
Crusted scabies	14 (3%)
**Severity of the skin lesion**	
Mild	13 (28%)
Moderate	191 (41%)
Severe	144 (31%)

**Table 4:** Scabies secondary bacterial infection rate by age, Amhara region, Ethiopia (2015).

Variable	Scabies Cases (*N*)	Infection (*N*)	Infection % (95% CI)	Odds Ratio	*p* Value
<2 years	46	7	15.2 (11.8–18.2)	1.8	0.012
2–18 years	288	101	35.1 (29.3–40.9)	4.2	<0.001
>18 years	132	11	8.3 (6.9–9.7)	Ref.	
Total	466	119	25.5 (21.3–29.7)		
